# The Ottawa Score Performs Poorly to Identify Cancer Patients at High Risk of Recurrent Venous Thromboembolism: Insights from the TROPIQUE Study and Updated Meta-Analysis

**DOI:** 10.3390/jcm11133729

**Published:** 2022-06-28

**Authors:** Corinne Frere, Benjamin Crichi, Clémentine Wahl, Elodie Lesteven, Jérôme Connault, Cécile Durant, Jose Antonio Rueda-Camino, Alexandra Yannoutos, Okba Bensaoula, Christine Le Maignan, Zora Marjanovic, Dominique Farge

**Affiliations:** 1 INSERM UMRS-1166, Institute of Cardiometabolism and Nutrition, GRC 27 GRECO, Sorbonne Université, F-75013 Paris, France; clementine.wahl@aphp.fr (C.W.); elodie.lesteven@aphp.fr (E.L.); 2 Internal Medicine Unit (UF 04): CRMR MATHEC, Maladies Auto-Immunes et Thérapie Cellulaire, Saint-Louis Hospital, Assistance Publique-Hôpitaux de Paris, F-75010 Paris, France; benjamin.crichi@aphp.fr (B.C.); christine.lemaignan@aphp.fr (C.L.M.); 3 Department of Internal Medicine, CHU de Nantes, F-44093 Nantes, France; jerome.connault@chu-nantes.fr (J.C.); cecile.durant@chu-nantes.fr (C.D.); 4 Department of Internal Medicine, Hospital Rey Juan Carlos, Móstoles, 28933 Madrid, Spain; jose.rueda@hospitalreyjuancarlos.es; 5 Vascular Medicine Department, Groupe Hospitalier Paris Saint-Joseph, F-75014 Paris, France; ayannoutsos@ghpsj.fr; 6 Institut Curie, F-92210 Saint-Cloud, France; okbaibn-nafaa.bensaoula@curie.fr; 7 Department of Hematology, Saint-Antoine Hospital, Assistance Publique-Hôpitaux de Paris, F-75012 Paris, France; zora.marjanovic@aphp.fr; 8 Institut Universitaire d’Hématologie, Université de Paris, EA 3518, F-75010 Paris, France

**Keywords:** cancer, venous thromboembolism, anticoagulants, recurrence, score

## Abstract

The Ottawa score (OS) for predicting the risk of recurrent venous thromboembolism (VTE) in cancer patients with VTE may help to guide anticoagulant treatment decisions that will optimize benefit-risk ratios. However, data on its reliability are conflicting. We applied the OS to all cancer patients with VTE enrolled in the prospective multicenter TROPIQUE study who received low-molecular-weight heparin over a 6-month period. Of 409 patients, 171 (41.8%) had a high-risk OS. The 6-month cumulative incidence of recurrent VTE was 7.8% (95%CI 4.2–14.8) in the high-risk OS group versus 4.8% (95%CI 2.6–8.9) in the low-risk OS group (SHR 1.47; 95%CI 0.24–8.55). The Area Under the Receiver Operating Characteristic curve (AUROC) of the OS in identifying patients who developed recurrent VTE was 0.53 (95%CI 0.38–0.65), and its accuracy was 57.9%. Among individual variables included in the OS, only prior VTE was significantly associated with the 6-month risk of recurrent VTE (SHR 4.39; 95% CI 1.13–17.04). When pooling data from all studies evaluating this score for predicting VTE recurrence in cancer patients (7 studies, 3413 patients), the OS estimated pooled AUROC was 0.59 (95%CI 0.56–0.62), and its accuracy was 55.7%. The present findings do not support the use of the OS to assess the risk of recurrent VTE in cancer patients.

## 1. Introduction

Monotherapy with low-molecular-weight heparins (LMWHs) has been the standard of care for the treatment of cancer-associated thrombosis (CAT) for three decades [1,2]. Six recent randomized-control trials (RCTs) compared direct oral anticoagulants (DOACs) with LMWHs in this clinical setting [3,4,5,6,7,8]. A pooled analysis of these RCTs reported that DOACs decreased the 6-month risk of recurrent venous thromboembolism (VTE) by 33% compared with LMWHs without increasing the risk of major bleeding [9]. However, a significant increase in the risk of clinically relevant non-major bleeding was observed [9]. Current clinical practices guidelines (CPGs) reviewed these new pieces of evidence and now recommend monotherapy with LMWHs or DOACs for at least 3–6 months as first-line treatment of VTE in medical oncology patients [10,11,12,13,14].

Weight-adjusted LMWHs with a reduction to 75% of the full-dose after the first month of anticoagulation remain the preferred option in selected cancer patients, including those at high risk of bleeding, those with gastrointestinal or genitourinary cancers, and those having a significant risk of drug-drug interactions (DDIs) [10,11,12,13,14]. However, for patients at high risk of recurrent VTE, LMWHs without dose reduction or DOACs may be a more appropriate first-line option. Effective clinical tools to assess individual risk of VTE recurrence are needed to guide anticoagulant treatment decisions that will optimize benefit-risk ratios.

The Ottawa score is currently the only risk assessment model (RAM) available to assess the risk of recurrent VTE in patients with CAT [15]. This simple point-based RAM incorporates five readily available clinical variables and can be used dichotomously to classify patients into high (sum score ≥1) versus low (sum score ≤0) risk for recurrent VTE. A previous meta-analysis of four studies applying the original Ottawa score (1558 patients) assessed its ability to discriminate between high- and low-risk patients [16]. The Ottawa score was reported to have an estimated pooled Area Under the Receiver Operating Characteristic curve (AUROC) of 0.7 (95% confidence interval (95% CI) 0.6–0.8), a sensitivity of 70% (95%CI 60–80), and a specificity of 50% (95%CI 50–60) [16]. Patients with a high-risk Ottawa score (49.3%) had a 6-month pooled crude rate of recurrent VTE of 18.6% (95% CI 13.9–23.9) compared to 7.4% (95%CI 3.4–12.5) for those with a low-risk Ottawa score [16]. However, this score failed to identify patients at high risk of recurrent VTE in two recent large prospective studies [17,18], thereby questioning its reliability.

Herein, we applied the original Ottawa score to all cancer patient with VTE enrolled in the multicenter, prospective, observational TROPIQUE study, which was conducted in 65 French centers involved in the care of cancer patients. This analysis aimed to evaluate the overall discriminatory performance of the Ottawa score in identifying patients with CAT at high risk of recurrent VTE while receiving long-term treatment with LMWHs. We also performed an updated systematic review and meta-analysis of all studies evaluating this score in external validation sets.

## 2. Materials and Methods

### 2.1. Study Design and Participants

Full details of the TROPIQUE study design have been published previously [19]. Briefly, patients were eligible if they: (i) were over 18 years old; (ii) had a histologically or cytologically confirmed diagnosis of solid or hematological cancer; (iii) were receiving anti-neoplastic treatment or palliative care; (iv) had an objectively diagnosed recent index VTE including symptomatic deep vein thrombosis (DVT) of the upper or lower limbs, pulmonary embolism (PE), visceral vein thrombosis (VVT), or central venous catheter (CVC)-related thrombosis; (v) were initiating long-term treatment with LMWHs according to current CPGs. The index VTE diagnosis was established by the referring physician based on the following objective standard routine clinical practice criteria: (i) for DVT: a non-compressible proximal or distal vein on compression ultrasonography; (ii) for PE: an intraluminal filling defect in one or more subsegmental or proximal pulmonary arteries on the spiral computed tomography (CT) scan; an intraluminal filling defect or a sudden cut-off of vessels more than 2.5 mm in diameter on the pulmonary angiogram; a perfusion defect of at least 75% of a segment with a local normal ventilation result (high probability) on ventilation/perfusion lung scintigraphy; (iii) for VVT: a thrombus detected on a (staging) abdominal or pelvic CT. Exclusion criteria were: (i) patients already treated with anticoagulants more than 7 days; (ii) any contraindication to LMWHs’ administration (hypersensitivity to LMWHs, active bleeding, previous heparin induced thrombocytopenia, severe renal impairment).

The study was approved by the Ile-de-France I Ethics Committee (Paris, France), and informed consent was obtained from all participants. The current report adheres to the TRIPOD checklist for Prediction Model Validation [20] and to the PROBAST tool on risk of bias and applicability in prediction model studies [21].

### 2.2. Data Collection and Study Outcomes

Demographic and clinical data, risk factors for VTE, and ongoing treatments were collected at study enrollment and during the 3- and 6-month follow-up visits. The Ottawa score was calculated at study entry, as previously described [15], based on five items: female sex (+1 point), lung cancer (+1 point), breast cancer (−1 point), local disease (i.e., cancer TNM stage I, −2 points), and prior VTE (+1 point). An Ottawa sum score ≤0 classified a patient as being at low risk for recurrent VTE, while an Ottawa sum score ≥1 classified a patient as being at high-risk for recurrent VTE [15].

For the present analysis, the primary outcome measure was recurrent symptomatic or incidental objectively confirmed VTE or VTE-related death within 6 months. Recurrent VTE was defined as objectively documented DVT of upper or lower limbs, PE, VVT or CVC-related thrombosis. All VTE events were adjudicated based on radiology reports. Patients were followed-up from inclusion until 6 months (end of follow-up) or earlier if death or lost to follow-up.

### 2.3. Statistical Analysis

Statistical analysis was performed using NCSS 2022 (NCSS LLC, Kaysville, UT, USA) and R (https://www.R-project.org (accessed on 28 February 2022) with the “cmprisk,” and “riskRegression” packages. All analyses were conducted on the intention-to-treat population (i.e., all included patients). Missing data were imputed using single imputation by predictive mean matching. Categorical variables were compared using the chi-square test or Fisher’s exact test, and continuous variables were compared using the Mann–Whitney test. The Fine & Gray competing risk model, considering non-VTE-related death as a competing risk [22], was used to estimate the cumulative incidences of recurrent VTE in the high-risk and low-risk Ottawa score groups with their corresponding 95% CI. The individual variables included in the Ottawa score were assessed by estimating the subdistribution hazard ratios (SHRs) with 95% CI at 6 months in a multivariable model including all score variables. The overall discriminatory performance of the continuous Ottawa score to predict recurrent VTE at the 6-month follow-up was assessed by calculating the Area Under the Receiver Operating Characteristic curve (AUROC, Efron C-index) and its 95% CI. The variable of interest was the continuous Ottawa score, and the dichotomous outcome variable was recurrent venous thromboembolism within 6 months.

All tests were 2-sided, and a *p*-value lower than 0.05 was considered as statistically significant.

### 2.4. Systematic Review and Pooled Analysis

We then performed a literature search using MEDLINE and EMBASE and the following key words: “Ottawa score” AND “recurrent venous thromboembolism” AND “cancer” from 1 June 2012 (online publication of the Ottawa score was 7 June 2012) to 19 March 2022. We used the Covidence software for systematic reviews (Melbourne, Australia) for records screening. Briefly, 2 reviewers (C.F. and B.C.) independently screened all records identified in the literature search for study eligibility based on title and abstract. Eligible studies evaluated the predictive ability of the original Ottawa score for recurrent VTE in cancer patients treated with any anticoagulant for an index VTE. Any discrepancies in study selection were resolved by consensus and adjudicated by a third author (D.F.). In case of duplicate publications, only the most recent publication was considered. The same 2 reviewers independently assessed study quality and extracted clinical and outcomes data using dedicated forms. The method of the inverse variance on the arcsine-transformed proportions (random effects model) was used to calculate the pooled rate of recurrent VTE in each level of clinical probability (high-risk and low-risk Ottawa score groups). Heterogeneity among studies was assessed using the Cochran Q statistic, and study consistency was quantified with the I^2^ statistics. Statistical analysis was performed using MetaXL (version 5.3).

## 3. Results

### 3.1. Performance of the Original Ottawa Score in the TROPIQUE Study Population

From November 2012 to August 2013, 409 out of 474 patients screened for eligibility were included in the TROPIQUE cohort at 65 participating centers in France (Appendix A) [19]. Patients’ baseline characteristics are summarized in Table 1. No patient was lost to follow-up. During the 6-month follow-up period, 19 patients developed recurrent VTE. Of these 19 patients, 5 (26.3%) developed isolated PE; 5 (26.3%) developed isolated DVT; 1 developed PE and DVT (5.3%); 6 (31.6%) developed isolated CVC-associated thrombosis; 1 developed DVT and CVC-associated thrombosis (5.3%); and 1 (5.3%) developed isolated VVT. Overall, the 6-month cumulative incidence of recurrent VTE was 6.2% (95% CI 4.0–9.5). Death from any cause occurred in 146 (35.79% (95% CI 31.05–40.34)) patients. Most deaths were related to cancer progression (87.5%).

**Figure 1:**
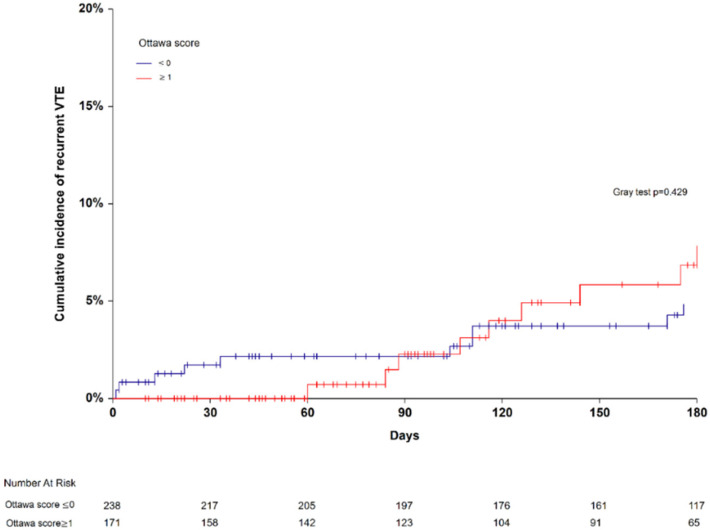
Six-month cumulative incidence of recurrent venous thromboembolism in patients with high- (≥1) and low-risk Ottawa score (≤0).

**Figure 2:**
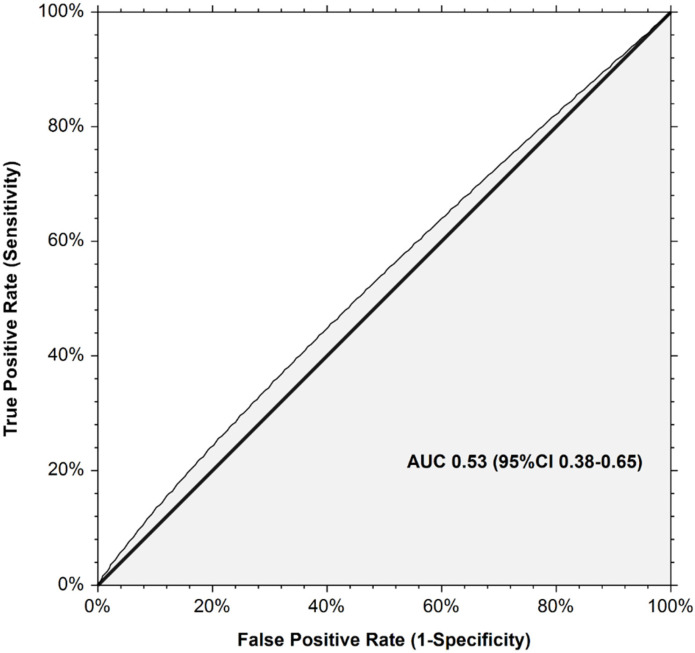
Receiver operating curve for the Ottawa score for prediction of recurrent venous thromboembolism in the TROPIQUE cohort.

At study enrollment, 171 patients (41.8% (95% CI 37.0–46.6)) were classified at high-risk for recurrent VTE and 238 (58.2% (95% CI 53.4–63.0)) patients at low risk. Nine recurrent VTE occurred in the high-risk Ottawa score group versus ten in the low-risk Ottawa score group. Six-month cumulative incidences of recurrent VTE did not significantly differ between the high-risk and the low-risk Ottawa score groups (7.8% (95% CI 4.2–14.8) versus 4.8% (95% CI 2.6–8.9), Gray test *p* = 0.429; SHR 1.47 (95% CI 0.24–8.55) in competing risk analysis, *p* = 0.670; Figure 1). The AUROC of the Ottawa score was 0.53 (95% CI 0.38–0.65; Figure 2). At the cutoff point defining high-risk (sum score ≥1), the model sensitivity was 47.4% (95% CI 24.4–71.1), and its specificity was 58.5.6% (95% CI 53.3–63.4). The corresponding positive and negative predictive values were 5.3% and 98.8%, respectively. The proportion of patients correctly classified (accuracy) was 57.9%. Excluding CVC-related thrombosis from the recurrent VTE events did not change the AUROC of the Ottawa score.

When evaluating the individual variables used in the Ottawa score in a multivariable model, only prior VTE was significantly associated with the 6-month risk of recurrent VTE (13.6% (95% CI 6.4–28.8) in patients with previous VTE versus 4.6% (95% CI 2.7–7.9) in patients without previous VTE, Gray test *p* = 0.020; SHR 4.39 (95% CI 1.13–17.04) in competing risk analysis, *p* = 0.033; Table 2 and Supplementary Figure S1). A classification based on previous VTE alone performed better than the Ottawa score in identifying patients who developed a recurrent VTE (AUROC 0.63 (95% CI 0.46–0.75); sensitivity 31.5% (95% CI 12.6–56.5); specificity 87.7% (95%CI 0.84–0.90); positive predictive value 11.1%; negative predictive value 96.3%; accuracy 85.1%). The 6-month cumulative incidence of recurrent VTE tended to be lower in women (3.3% (95% CI 1.4–7.9)) than in men (8.2% (95% CI 5.0–13.7), Gray test *p* = 0.05871; SHR 0.50 (95%CI 0.16–1.52); Supplementary Figure S1 and Table S1). The 6-month cumulative incidence of recurrent VTE by primary tumor site is shown in Supplementary Figure S1 and Table S1 and tended to be higher in patients with lung (12.1% (95% CI 3.7–39.9)) and genitourinary (11.2% (95% CI 5.2–24.1)) cancers compared to other cancers. Finally, the 6-month cumulative incidence of recurrent VTE tended to be higher in patients with metastatic cancer (7.6% (95% CI 4.6–12.8)) compared to those with localized cancer (4.5% (95% CI 1.7–11.9), Gray test *p* = 0.28339; Supplementary Figure S1 and Table S1).

### 3.2. Pooled Analysis of Studies That Evaluated the Original Ottawa Score in Predicting CAT Recurrence

**Figure 3:**
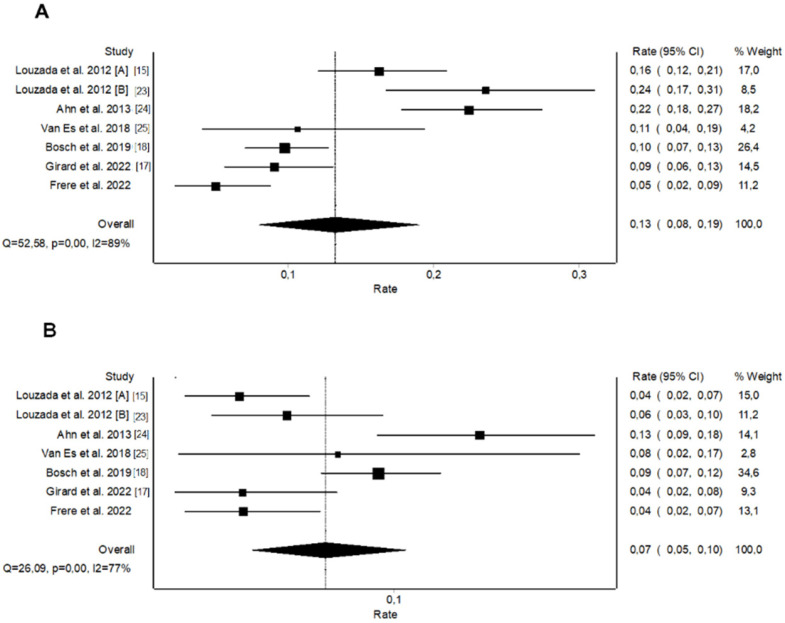
Pooled rates of recurrent venous thromboembolism for the original Ottawa score: (**A**) High-risk patients, (**B**) Low-risk patients.

We performed a systematic review and pooled analysis of all studies that evaluated the dichotomized original Ottawa score in predicting recurrent VTE in patients with CAT. The literature search identified 102 potentially relevant citations. Sixteen records were duplicates; 78 were excluded after title and abstract screening; and 12 were assessed for eligibility (Supplementary Figure S2). Six studies meeting the inclusion criteria [15,17,18,23,24,25] were added to the present post-hoc analysis of the TROPIQUE study resulting in a pooled analysis of 3413 patients (Supplementary Table S2). Patients with a high-risk Ottawa score (46.7%) had a pooled 6-month rate of recurrent VTE of 13.2% (95% CI 8.5–18.7%; I^2^ = 89%; *p* < 0.001, Figure 3) versus 6.8% (95% CI 4.4–9.6%; I^2^ = 77%; *p* < 0.001, Figure 3) for those with a low-risk Ottawa score. The dichotomized Ottawa score had an estimated pooled AUROC of 0.59 (95% CI 0.56–0.62), with a sensitivity of 61.5% (95% CI 56.2–66.6), a specificity of 55.0% (95% CI 53.2–56.8), and an accuracy of 55.7%. When restricting the analysis to prospective studies including more than 200 patients, the pooled 6-month rate of recurrent VTE was 8.2% (95% CI 5.6–11.1%; I^2^ = 50%; *p* = 0.14) in patients with a high-risk Ottawa score versus 5.9% (95% CI 2.7–10.0%; I^2^ = 80%; *p* = 0.01) in those with a low-risk Ottawa score (Supplementary Figure S3). The corresponding estimated pooled AUROC was 0.53 (95% CI 0.48–0.57), with a sensitivity, specificity, and accuracy of 48.6% (95% CI 40.4–57.0), 55.82% (95% CI 53.4–58.2), and 55.2%, respectively.

## 4. Discussion

The Ottawa score is currently the only RAM available for predicting the risk of VTE recurrence in cancer patients. Developed by Louzada et al., in 2012 [15], this score has been assessed in several external validation studies with conflicting results [17,18,23,24,25]. Applying the Ottawa score to cancer patients enrolled in the prospective TROPIQUE study who were treated with LMWHs for a confirmed index VTE failed to identify accurately those who developed recurrent VTE within 6 months, as reflected by an AUROC of 0.53 and an accuracy of 57.9%.

Our results are in line with those from the recent prospective PREDICARE study [17]. In this cohort of 409 patients with CAT who received long-term treatment with LMWHs, the Ottawa score did not identify those who developed recurrent VTE within 6 months, as reflected by an AUROC of 0.60 (95% CI 0.55–0.65), a sensitivity of 75.0% (95% CI 55.1–89.3), and a specificity of 43.3% (95% CI 38.2–48.5). The original Ottawa score was initially derived to discriminate patients with an a priori risk of VTE recurrence under anticoagulation <7% from those with an a priori risk for VTE recurrence ≥7% [15]. In the TROPIQUE and PREDICARE studies, the rates of recurrent VTE were lower than in previous studies, i.e., 4.6% and 7.0% for TROPIQUE and PREDICARE, respectively, compared to approximately 10% in previous validation studies [16].

Similarly, in a post-hoc analysis of the HOKUSAI-VTE CANCER trial [18], which included 1046 patients with CAT receiving long-term treatment with either dalteparin or edoxaban, the Ottawa score had an overall AUROC of 0.52 (95% CI 0.46–0.58). The risk of recurrent VTE was 9.8% in patients with a high-risk Ottawa score compared to 9.4% in those with a low-risk Ottawa score (corresponding to a SHR of 1.20 (95% CI, 0.81–1.80)). A similar poor discriminatory performance was observed in both the dalteparin (AUROC 0.49 (95% CI, 0.42–0.57)) and the edoxaban groups (AUROC 0.55 (95% CI, 0.46–0.64)). When pooling the results from these three prospective studies (TROPIQUE, PREDICARE [17], HOKUSAI-VTE CANCER [18]), the estimated AUROC, sensitivity, specificity, and accuracy of the Ottawa score were 0.53 (95%CI 0.48–0.57), 48.6% (95%CI 40.4–57.0), 55.82% (95%CI 53.4–58.2), and 55.2%, respectively. Differences in study design (prospective versus retrospective), clinical setting, geographical locations, case-mix, follow-up periods, treatment regimens, and overall rates of recurrent VTE across cohorts may partly explain why these findings are inconsistent with previous validation studies [18,23,24,25]. Furthermore, most of these validation studies, except PREDICARE [17], did not consider the competing risk of death, which may have led to an overestimation of differences in risk estimates across high and low Ottawa score groups.

Numerous factors may influence the overall risk of recurrent VTE in patients with CAT. The original Ottawa score incorporates five items including female sex, lung cancer, breast cancer, cancer stage I, and prior VTE. In the present study, when these variables were evaluated individually in a multivariable model, only prior VTE was significantly associated with the risk of recurrent VTE.

The original Ottawa score derivation study reported that female sex was associated with a trend towards a lower risk of recurrent VTE (SHR 0.50 (95% CI 0.14–1.52)). Data regarding gender differences in CAT outcomes are conflicting. A retrospective analysis of the RIETE registry comparing the rates of recurrent VTE, major bleeding, and mortality in 5104 women and 5951 men with CAT did not report any gender difference in the rates of recurrent PE or DVT [26]. On the contrary, a recent analysis of the international, non-interventional PREFER in the VTE registry of patients with a first episode of acute symptomatic VTE reported that the rates of recurrent VTE within 12 months were higher in women with cancer (17.6%) compared to men with cancer (9.1%), with an absolute difference of 8.6% (95% CI 2.5–19.7%) [27].

It has been widely demonstrated that the site of primary cancer is a major determinant of the risk of developing a first CAT, but it is also associated with the risk of recurrent VTE. A post hoc analysis of the CLOT trial [28] first highlighted that lung cancer was associated with a significantly higher risk of recurrent VTE (HR 3.51, 95% CI 1.62–7.62) compared to other cancer types, while breast cancer tended to be associated with a lower risk (HR 0.59, 95% CI, 0.17–2.01). In the Ottawa score derivation study, lung and breast cancers were significantly associated with the risk of recurrent VTE in multivariate analysis. Consequently, presence of a lung cancer adds one point to the overall Ottawa score, while a breast cancer removes one point. A retrospective analysis of 3947 cancer patients included in the RIETE registry reported that the rate of recurrent VTE was 27 events per 100 patient-years (95% CI 22–33) in patients with lung cancer compared to 5.6 events per 100 patient-years (95% CI 3.8–8.1) in patients with breast cancer [29]. Similar to the results of the recent PREDICARE prospective study [17], we found that neither lung cancer nor breast cancer was significantly associated with the risk of recurrent VTE. We observed a trend towards a higher 6-month cumulative incidence of recurrent VTE in patients with lung (12.1% (95% CI 3.7–39.9)) and genitourinary (11.2% (95% CI 5.2–24.1)) cancers as compared to those with breast cancer (1.54% (95% 0.22–10.76)). Interestingly, a recent post hoc analysis of the CARAVAGGIO study found that the absolute risk difference in recurrent VTE in favor of apixaban was 5.5% in patients with lung cancer, 3.7% in those with genitourinary cancer, and 0.15% in those with breast cancer, suggesting that DOACs may be a more efficient option in lung cancer, provided the patients did not have a high risk of bleeding or of DDIs [30].

In the TROPIQUE study, metastatic cancer was associated with a trend towards a higher risk of recurrent VTE compared to locally advanced or localized cancers. A recent post-hoc analysis of the CARAVAGGIO study showed that patients with locally advanced (HR 2.8, 95% CI 1.1–6.9) and metastatic cancer (HR 3.3, 95% CI 1.4–7.7) have a higher rate of VTE recurrence than those with localized cancer [31]. However, any anticoagulant type should be used with caution in patients with metastases since they are at high risk of major bleeding [31].

With DOACs or LMWHs as possible first-line options for the treatment of CAT, clinicians are now faced with more complex anticoagulant treatment decisions. DOACs or a full dose of LMWHs can be used throughout the first 6 months of treatment when the risk of recurrent VTE is high, while in those with a low risk of recurrent VTE, dose de-escalation of LMWHs (75–80% of full dose) after 1 month, or Vitamin K antagonists (VKAs), may be more appropriate. Our data suggest that the Ottawa score does not provide sufficient predictive reliability to guide clinical decision making. Therefore, a personalized approach, based on individual patient risk factors, a benefit-risk ratio of each drug, physician’s judgment, and patient values and preferences, remains essential in optimizing anticoagulant treatment decisions.

Major limitations of the present study relate to the population sample size and to the relatively small number of recurrent VTE events observed during the 6-month follow-up (4.6%). Since the TROPIQUE study was not initially designed to validate the Ottawa score [19], there was no sample size calculation for the present analysis. However, in the PREDICARE study [17], which was specifically designed to validate the Ottawa score in patients with CAT receiving LMWHs for 6 months, the calculated sample size was at least 392 patients to validate the score with an expect AUROC of 0.70 with a lower limit of its CI of 0.65. The TROPIQUE cohort included 409 patients [19].

## 5. Conclusions

The present findings do not support the use of the Ottawa score to personalize the treatment of CAT during the first 6 month of anticoagulant therapy. A multidisciplinary patient-centered approach, with close cooperation between oncologists and other specialists, balancing risk of benefit and harm for each individual patient, and taking account of patient values and preferences, is required to optimize anticoagulant therapy. According to current CPGs [10,11,12,13,14], anticoagulant treatment should be reassessed on a regular basis throughout the course of the disease and continued as long as the cancer is active.

## Figures and Tables

**Table 1:** Baseline characteristics of patients included in the TROPIQUE study.

Patient Characteristics	All (*n* = 409)	Low-Risk Ottawa Score	High-Risk Ottawa Score	*p*
		(*n* = 238)	(*n* = 171)	
**Age (years), mean ± SD**	65.0 ± 12.1	63.5 ± 12.9	65.9 ± 10.8	ns
**Women, no. (%)**	204 (49.8)	90 (35.6)	114 (73.1)	<0.0001
**BMI (kg/m^2^), mean ± SD**	24.8 ± 5.1	25.2 ± 4.9	24.2 ± 5.3	0.0052
**ECOG > 2, no. (%)**	49 (11.9)	23 (9.7)	26 (17.1)	ns
Missing data	3	2	1	
**Estimated GFR, no. (%)**				ns
<60 mL/min/1.73 m^2^	65 (16.7)	34 (15.4)	31 (20.5)	
Missing data	22	17	5	
**Cancer type, no. (%)**				
Gastrointestinal	100 (24.4)	60 (25.2)	40 (23.4)	ns
Breast	65 (15.9)	57 (23.9)	8 (4.7)	<0.0001
Lung	71 (17.4)	7 (2.9)	64 (37.4)	<0.0001
Hematological	54 (13.2)	46 (19.3)	8 (4.7)	<0.0001
Genitourinaty	38 (9.3)	30 (12.6)	8 (4.7)	0.0088
Other cancers	81 (19.8)	38 (16.0)	43 (25.1)	0.0239
**Cancer Stage, no. (%)**				
Stage I	97 (23.7)	97 (40.8)	0 (0)	<0.0001
Stage II	61 (14.9)	29 (12.2)	32 (18.7)	ns
Stage III–IV	251 (61.4)	112 (47.1)	139 (81.3)	<0.0001
Ongoing cancer treatment at time of diagnosis *, no. (%)				
Chemotherapy	328 (80.2)	186 (78.2)	142 (83.0)	ns
Hormonal therapy	26 (6.4)	16 (6.7)	10 (5.8)	ns
Radiotherapy	37 (9.0)	24 (10.1)	13 (7.6)	ns
Antiangiogenics	22 (5.4)	13 (5.5)	9 (5.3)	ns
Targeted therapy	53 (13.0)	34 (14.3)	19 (11.1)	ns
Supportive care	32 (7.8)	17 (7.1)	15 (8.8)	ns
**Risk factors for VTE, no. (%)**				
Prior VTE	54 (13.2)	17 (7.1)	37 (21.6)	<0.0001
Major surgery in previous month	100 (24.4)	68 (28.6)	32 (18.7)	0.0265
CVC	303 (74.1)	179 (75.2)	124 (72.5)	ns
Immobilization in previous month	47 (11.5)	23 (9.7)	24 (14)	ns
Thrombophilia	6 (1.5)	5 (2.1)	1 (0.6)	ns
Index VTE *, no. (%)	145 (35.5)	75 (31.5)	70 (40.9)	0.0264
PE	193 (47.2)	112 (47.1)	81 (47.4)	ns
DVT of the lower limb	45 (11.0)	28 (11.8)	17 (9.9)	ns
DVT of the upper limb	16 (3.9)	11 (4.6)	5 (2.9)	ns
Visceral vein thrombosis	66 (16.1)	45 (18.9)	21 (66)	ns
CVC-related thrombosis				

* One or more. Abbreviations: BMI, body mass index; CVC, central venous catheter; DVT, deep vein thrombosis; GFR, glomerular filtration rate; ns, not significant; PE, pulmonary embolism; VTE, venous thromboembolism.

**Table 2:** Multivariable analyses for recurrent VTE during the 6-month follow-up.

Variables Included in the Ottawa Score		SHR (95% CI)	*p*-Value
**Sex**		-	
	Men	Ref	
	Women	0.499 (0.164–1.52)	0.220
**Lung cancer**		-	
	No	Ref	
	Yes	2.172 (0.4296–10.98)	0.350
**Breast**		-	
	No	Ref	
	Yes	0.469 (0.0397–5.55)	0.550
**TNM Stage 1**		-	
	No	Ref	
	Yes	0.653 (0.1704–2.50)	0.530
**Prior venous thromboembolism**		-	
	No	Ref	
	Yes	4.395 (1.1300–17.09)	0.033

## Data Availability

The data that support the findings of this study are available from the corresponding author, C.F. and D.F., upon reasonable request.

## Supplementary Materials

The following supporting information can be downloaded at: https://www.mdpi.com/article/10.3390/jcm11133729/s1, Figure S1: Six-month cumulative incidence of recurrent VTE according to sex, primary tumor site, cancer stage, and previous VTE; Figure S2: PRISMA Flow diagram for systematic review study selection; Figure S3: Pooled recurrence rates of recurrent VTE for the original Ottawa score in prospective studies including more than 200 patients; Table S1: Six-month cumulative incidence of recurrent VTE according to primary tumor site; Table S2: Characteristics of studies included in the pooled analysis; Table S3: TRIPOD Checklist: Prediction Model Validation.

## Appendix A

**TROPIQUE investigators:** F. Abbadie, E. Achille, S. Ahmed, A. Alibay, S. Antoun, S. Aquilanti, R. Belhadj Chaidi, C. Belizna, L. Bengrine-Lefevre, Y. Benhamou, H. Bennami, O. Bensaoula, J.F. Berdah, I. Bonnet, A. Bura Riviere, F. Cajfinger, J. Camuset, G. Catimel, A. Cattey- Javouhey, N. Caunes, O. Chiche, O. Cojocarasu, J. Connault, J. Constans, P. Cony-Makhoul, M. Coudurier, F. Couturaud, S. Culine, J. Dauba, F. Del Piano, S. Dennetiere-Cluet, C. Desauw, A. Desplechin, L.M. Dourthe, C. Durant, B. Duvert, B. El sayadi, J. Ezenfis, C. Fabre, L. Falchero, N. Falvo, D. Farge, E. Ferrari, J.P. Fricker, A. Frikha, M.L. Garcia, G. Gerotziafas, F. Ghiringhelli, P. Girard, P. Goffette, A. Grenot-Mercier, A. Hamade, M. Hebbard, R. Hervé, L. Karlin, A. Khau Van Kien, P. Lanoye, S. Le Roy, F. Lecomte, G. Leftheriotis, E. Legac, I. Mahé, F. Maloisel, B. Maneglia, N. Mathe, B. Mennecier, G. Meyer, L. Moreau, M. Mousseau, M. Nae-Rejo, S. Nahon, H. Paradis, D. Péré-Veré, B. Peyrachon, G. Peyre, D. Pouessel, C. Pricaz, C. Quashie, S. Regina, C. Roncato, R. Rosario, S. Sadot-Lebouvier, A. Scherpereel, J. Schmidt, M.A. Sevestre, D. Spaeth, D. Stephan, D. Tavan, L. Vedrine, J.M. Vernejoux.
