# Supplementary material for: The Ottawa Score Performs Poorly to Identify Cancer Patients at High Risk of Recurrent Venous Thromboembolism: Insights from the TROPIQUE Study and Updated Meta-Analysis
Source: J Clin Med. 2022 Jun 28;11(13):3729. doi: 10.3390/jcm11133729 (PMC9267563; doi:10.3390/jcm11133729)
Supplement: Supplementary file 1 [file jcm-11-03729-s001.zip › jcm-1759730-supplementary.pdf]

Supplementary material

Figure S1. Six-month cumulative incidence of recurrent venous thromboembolism according to (A) sex, (B) primary tumor site, (C) cancer stage, and (D) previous VTE.

A

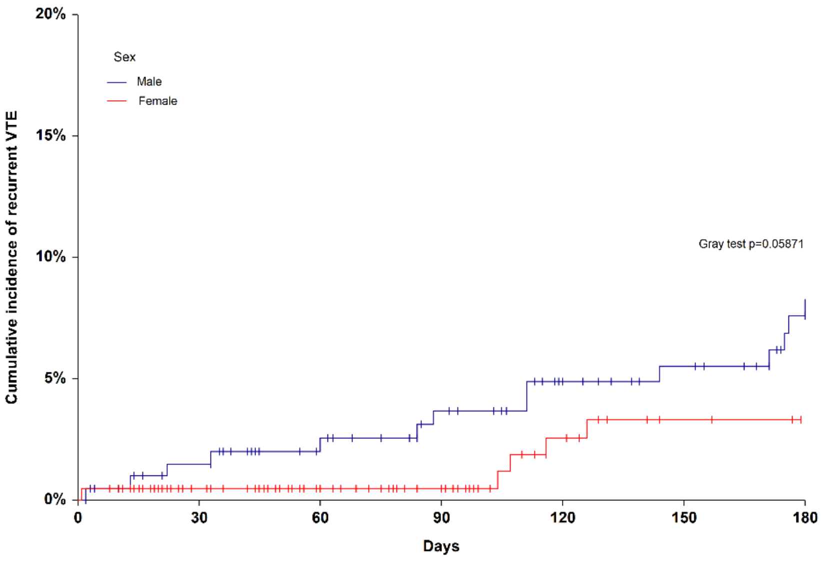

Number at Risk

Male	205	193	181	167	147	135	95
Female	204	182	166	153	133	117	87

B

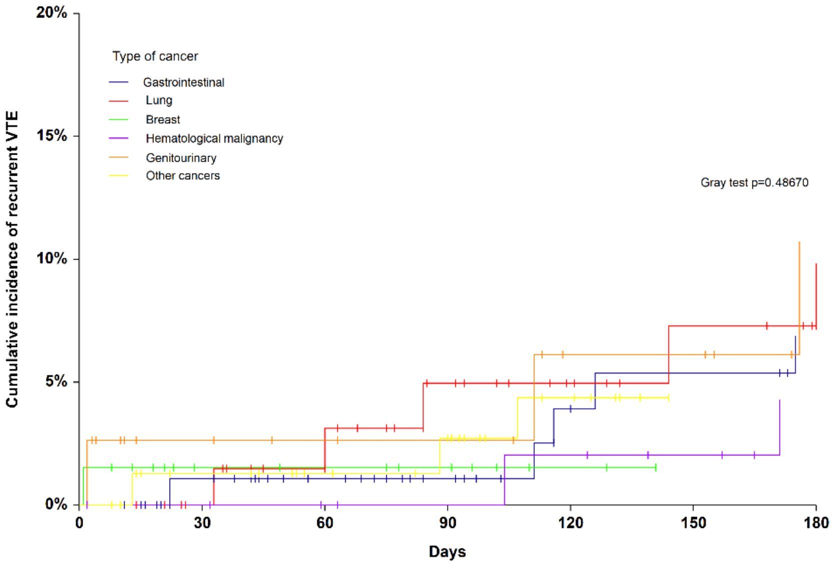

Number At Risk

Gastrointestinal	100	90	81	74	63	58	42
Lung	71	67	60	50	44	37	27
Breast	65	58	57	54	50	47	41
Hematological malignancy	54	53	50	48	45	42	28
Genitourinary	38	32	30	29	23	23	14
Other cancers	81	75	69	65	55	45	30

C

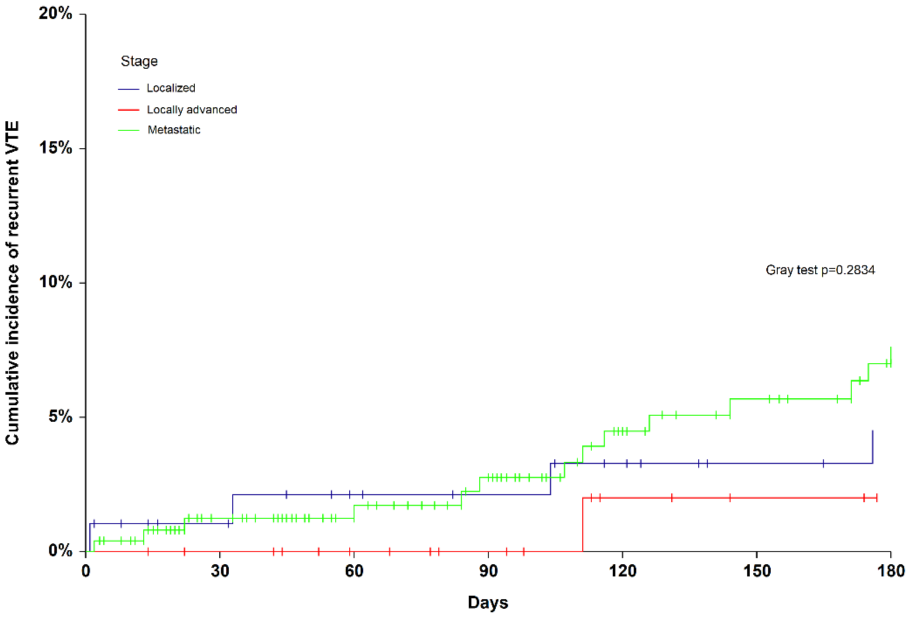

Number at Risk

Localized	97	92	87	84	78	69	50
Locally advanced	61	59	55	51	46	42	30
Metastatic	251	224	205	185	156	141	102

D

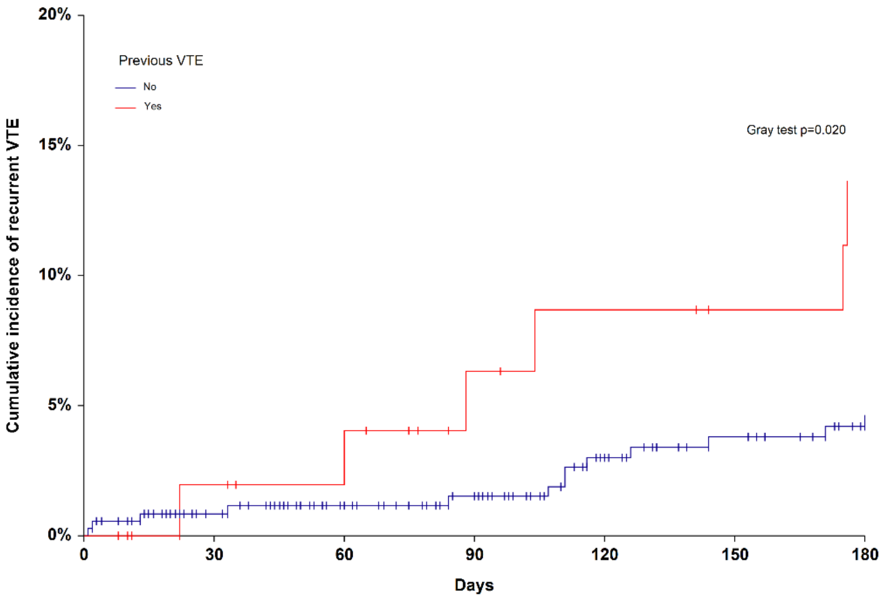

Number at Risk

No previous VTE	355	326	300	279	242	217	153
Previous VTE	54	49	47	41	38	35	29

Table S1. Six-month cumulative incidence of recurrent venous thromboembolism according to primary tumor site in the prospective TROPIQUE cohort.

Primary sites	Number of patients	Six-month cumulative incidence of recurrent VTE (95% CI)
Gastrointestinal	100	6.89% (2.94-16.16)
Breast	65	1.54% (0.22-10.76)
Lung	71	9.85% (4.19-23.15)
Haematological	54	4.28% (1.10-16.66)
Genitourinaty	38	10.70% (3.55-32.24)
Other cancers	81	4.37% (1.43-13.29)

Figure S2. PRISMA Flow diagram for systematic review study selection

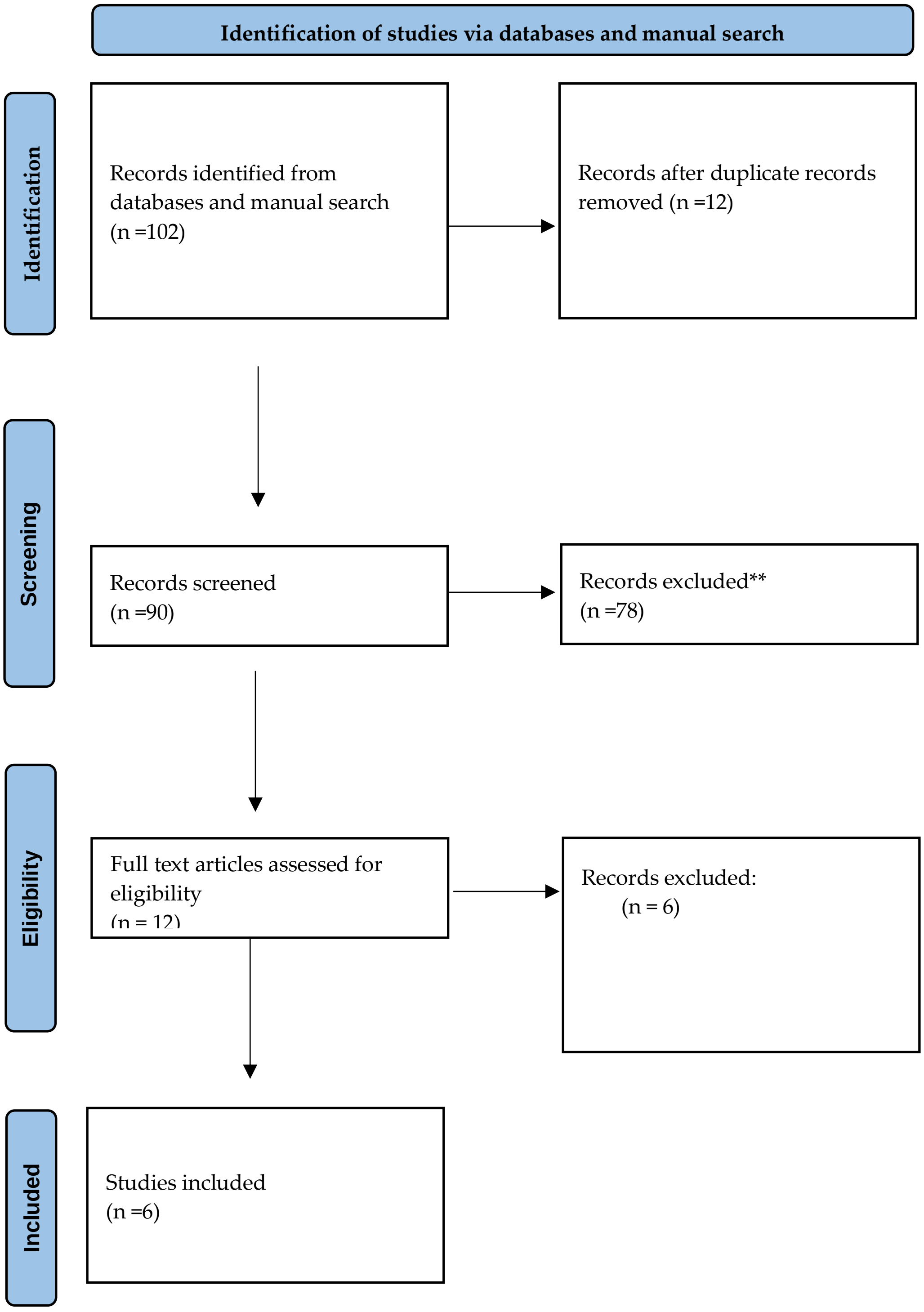

Table S2. Characteristics of studies included in the pooled analysis.

Study	Design	Setting	Inclusion period	n	Female sex	Lung cancer	Breast Cancer	Previous VTE	Rates of recurrent VTE
Louzada et al. 2012 [A] [9]	Retrospective	Single-center	2002-2004	543	55.8%	17.7%	15.6%	8.5%	10.1%
Louzada et al. 2012 [B] [17]	Retrospective	Multicenter	2006-2011	353	57.7%	17.5%	11%	21.8%	12.5%
Ahn et al. 2012[18]	Retrospective	Single-center	2007-2010	546	53.8%	17.2%	6.6%	4.8%	18.1%
Van Es et al 2018[19]	Prospective	Multicenter	2012-2014	117	50.4%	22.2%	8.5%	6.3%	9.4%
Girard et al.2022 [11]	Prospective	Multicenter	2015-2016	409	48.7%	31.3%	13.9%	12%	7.0%
Bosch et al. 2019[12]	Prospective	Multicenter	2015-2016	1046	48.3%	14.5%	11.8%	10.7%	9.5%
Frere et al. 2022	Prospective	Multicenter	2012-2013	409	49.8%	17.4%	15.9%	13.2%	4.6%

Figure S3. Pooled recurrence rates of recurrent venous thromboembolism for the original Ottawa score in prospective studies including more than 200 patients. (A) High-risk patients. (B) Low-risk patients.

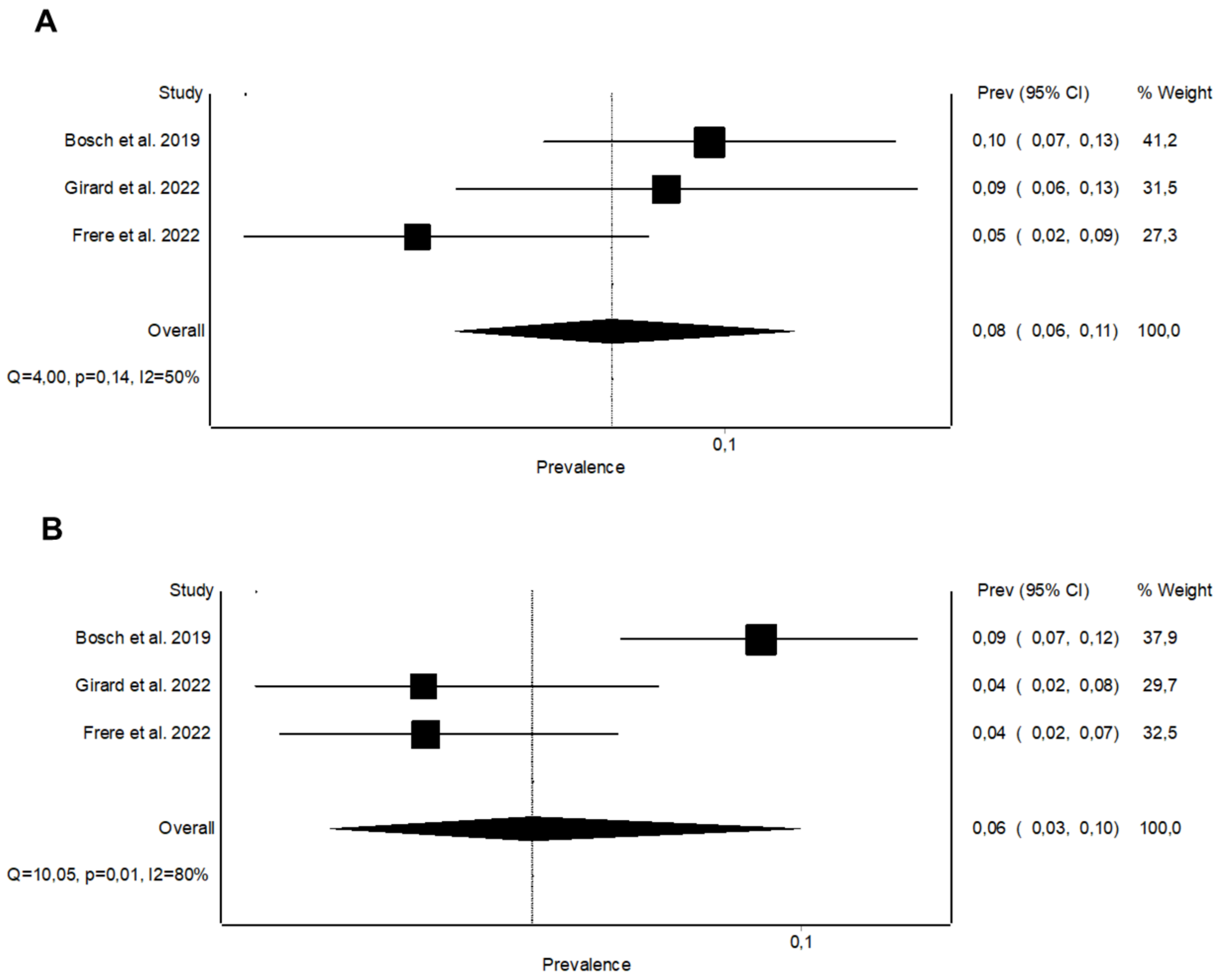

Table S3. TRIPOD Checklist: Prediction Model Validation

Section/Topic		Checklist Item	Page
**Title and abstract**			
Title	1	Identify the study as developing and/or validating a multivariable prediction model, the target population, and the outcome to be predicted.	1
Abstract	2	Provide a summary of objectives, study design, setting, participants, sample size, predictors, outcome, statistical analysis, results, and conclusions.	1
**Introduction**			
Background and objectives	3a	Explain the medical context (including whether diagnostic or prognostic) and rationale for developing or validating the multivariable prediction model, including references to existing models.	1-2
	3b	Specify the objectives, including whether the study describes the development or validation of the model or both.	2
**Methods**			
Source of data	4a	Describe the study design or source of data (e.g., randomized trial, cohort, or registry data), separately for the development and validation data sets, if applicable.	2-3
	4b	Specify the key study dates, including start of accrual; end of accrual; and, if applicable, end of follow-up.	2-3
Participants	5a	Specify key elements of the study setting (e.g., primary care, secondary care, general population) including number and location of centres.	2-3
	5b	Describe eligibility criteria for participants.	2-3
	5c	Give details of treatments received, if relevant.	2-3
Outcome	6a	Clearly define the outcome that is predicted by the prediction model, including how and when assessed.	2-3
	6b	Report any actions to blind assessment of the outcome to be predicted.	2-3
Predictors	7a	Clearly define all predictors used in developing or validating the multivariable prediction model, including how and when they were measured.	2-3
	7b	Report any actions to blind assessment of predictors for the outcome and other predictors.	2-3
Sample size	8	Explain how the study size was arrived at.	2-3
Missing data	9	Describe how missing data were handled (e.g., complete-case analysis, single imputation, multiple imputation) with details of any imputation method.	2-3
Statistical analysis methods	10c	For validation, describe how the predictions were calculated.	2-3
	10d	Specify all measures used to assess model performance and, if relevant, to compare multiple models.	2-3
	10e	Describe any model updating (e.g., recalibration) arising from the validation, if done.	-
Risk groups	11	Provide details on how risk groups were created, if done.	2-3
Development vs. validation	12	For validation, identify any differences from the development data in setting, eligibility criteria, outcome, and predictors.	2-3
**Results**			
Participants	13a	Describe the flow of participants through the study, including the number of participants with and without the outcome and, if applicable, a summary of the follow-up time. A diagram may be helpful.	4
	13b	Describe the characteristics of the participants (basic demographics, clinical features, available predictors), including the number of participants with missing data for predictors and outcome.	4
	13c	For validation, show a comparison with the development data of the distribution of important variables (demographics, predictors and outcome).	9
Model performance	16	Report performance measures (with CIs) for the prediction model.	4-8
Model-updating	17	If done, report the results from any model updating (i.e., model specification, model performance).	-
**Discussion**			
Limitations	18	Discuss any limitations of the study (such as nonrepresentative sample, few events per predictor, missing data).	9-10
Interpretation	19a	For validation, discuss the results with reference to performance in the development data, and any other validation data.	9-10
	19b	Give an overall interpretation of the results, considering objectives, limitations, results from similar studies, and other relevant evidence.	9-10
Implications	20	Discuss the potential clinical use of the model and implications for future research.	10
**Other information**			

Supplementary information	21	Provide information about the availability of supplementary resources, such as study protocol, Web calculator, and data sets.	appendix
Funding	22	Give the source of funding and the role of the funders for the present study.	11
